# Intensive chemotherapy for acute myeloid leukemia differentially affects circulating T_C_1, T_H_1, T_H_17 and T_REG _cells

**DOI:** 10.1186/1471-2172-11-38

**Published:** 2010-07-09

**Authors:** Elisabeth Ersvaer, Knut Liseth, Jørn Skavland, Bjørn Tore Gjertsen, Øystein Bruserud

**Affiliations:** 1 Institute of Medicine, University of Bergen, Bergen, Norway; 2 Department of Medicine, Haukeland University Hospital, Bergen, Norway

## Abstract

**Background:**

Several observations suggest that immunological events early after chemotherapy, possibly during the period of severe treatment-induced cytopenia, are important for antileukemic immune reactivity in acute myeloid leukemia (AML). We therefore investigated the frequencies of various T cell subsets (T_C_1, T_H_1, T_H_17) and CD25^+ ^FoxP3^+ ^T_REG _cells in AML patients with untreated disease and following intensive chemotherapy.

**Results:**

Relative levels of circulating T_C_1 and T_H_1 cells were decreased in patients with severe chemotherapy-induced cytopenia, whereas T_H_17 levels did not differ from healthy controls. Increased levels of regulatory CD25^+ ^FoxP3^+ ^T cells were detected in AML patients with untreated disease, during chemotherapy-induced cytopenia and during regeneration after treatment. T_H_17 and T_H_1 levels were significantly higher in healthy males than females, but this gender difference was not detected during chemotherapy-induced cytopenia. Finally, exogenous IL17-A usually had no or only minor effects on proliferation of primary human AML cells.

**Conclusions:**

We conclude that the effect of intensive AML chemotherapy differ between circulating T cell subsets, relative frequencies of T_H_17 cells are not affected by chemotherapy and this subset may affect AML cells indirectly through their immunoregulatory effects but probably not through direct effects of IL17-A.

## Background

Acute myeloid leukemia (AML) is characterized by clonal expansion of immature myeloblasts in the bone marrow and eventually leukemization [1]. The only curative treatment is intensive chemotherapy that can be combined with stem cell transplantation especially in younger patients below 60-65 years of age [1]. This treatment is followed by a period of severe bone marrow failure with severe pancytopenia, including lymphopenia. Rapid lymphoid reconstitution after therapy is associated with increased AML-free survival, an observation strongly suggesting that immunological events early after chemotherapy are clinically important [2,3]. This association between reconstitution and survival has been observed after conventional intensive chemotherapy [4], autotransplantation [5] and allotransplantation [6,7]. The mechanisms behind these associations are not known but may involve (i) treatment-induced immunogenic cell death with translocation of endo-calreticulin to the cell surface and induction of antileukemic T cell reactivity [8], (ii) increased efficiency of antileukemic immune reactivity during this period of low leukemia cell burden, or (iii) treatment-induced alterations of immunoregulatory networks. The remaining lymphocytes during chemotherapy-induced cytopenia may thus be important for this antileukemic effect.

Previous studies have described increased levels of circulating immunosuppressive CD4^+ ^CD25^HIGH ^T_REG _cells for patients with untreated AML [9,10] and these increased levels persist after chemotherapy when complete hematological remission is achieved [9]. The pretreatment T_REG _levels even seem to predict the response to the chemotherapy [9]. The peripheral blood levels of proinflammatory T_H_17 cells and IL17 plasma levels are also suggested increased in untreated AML, but in contrast to T_REG _cells these levels normalize when complete remission is achieved [11]. However, the levels of various T cell subsets during the early period of chemotherapy-induced cytopenia have not been investigated previously, and in the present study we therefore compared the T cell subset distribution for AML patients (i) before treatment, (ii) during the period of severe chemotherapy-induced cytopenia, and (iii) during hematopoietic reconstitution after treatment. The T cell subsets investigated were IFNγ secreting CD8^+ ^cytotoxic T (T_C_1) and CD4^+ ^helper T (T_H_1) cells, IL17-A secreting CD4^+ ^helper (T_H_17) cells and CD4^+ ^CD25^+ ^FoxP3^+ ^regulatory T (T_REG_) cells.

## Methods

### Patients

The studies were approved by the local Ethics Committee (Regional Ethics Committee III, University of Bergen, Bergen, Norway). All blood samples were collected after informed consent. AML was diagnosed according to the WHO criteria [12].

#### Patients with newly diagnosed AML

In our studies of primary human AML cells we included unselected patients with high peripheral blood blast counts; enriched leukemic cell populations could then be prepeared by density gradient separation alone (see below).

#### AML patients receiving intensive chemotherapy

Samples for analysis of T cell subsets were obtained from 20 patients receiving intensive cytarabine-based therapy (referred to as the first cohort). All patients were sampled between 0730 and 0900 a.m., and the analysis of samples or preparation of cultures started within 3 hours after sampling. These patients represent a consecutive group admitted to our hospital for either induction or consolidation therapy. For all patients receiving induction chemotherapy (i.e. patients with detectable AML prior to treatment) examination of bone marrow samples drawn during cytopenia 14 days after start of therapy verified that leukemic cells could no longer be detected; these patients were only included after the bone marrow control. The other patients were tested following consolidation therapy, i.e. they had achieved disease control and were in complete hematological remission before treatment. Thus, none of the patients had detectable disease when tested during cytopenia. Most of our patients (n = 16; median age 57.5 years with range 30-64 years; 5 males and 11 females) were investigated during the period of severe chemotherapy-induced cytopenia with peripheral blood neutrophils < 0.5 × 10^9^/L and dependency on regular platelet transfusion to maintain blood platelets > 10-20 × 10^9^/L. The last four patients were only investigated during the regeneration after cytopenia. The patients tested during pancytopenia were also lymphopenic with total lymphocyte counts in peripheral blood < 0.5 × 10^9^/L.

Six patients in this first group (median age 55.5 years with range 39-64 years; 2 males and 4 females) were examined during hematopoietic reconstitution, i.e. within 10 days after stable peripheral blood neutrophils > 0.5 × 10^9^/L. Four of these patients had reached hematological remission prior to sampling (i.e. they received consolidation chemotherapy); the last two patients reached complete remission within one week after sampling (i.e. they received induction chemotherapy).

We also analyzed the peripheral blood levels of various T cell subsets in a second group of 8 patients that represent a consecutive group investigated during a one month period (median age 50 years with range 17-67 years; 5 males and 3 females). This group is referred to as the second cohort. The absolute as well as the relative levels of various T cell subsets were investigated before (only available for 6 patients), during and following chemotherapy-induced cytopenia for the patients; paired statistical analyses were thereby possible.

Two additional groups with cytopenic patients were also examined. Firstly, we used a whole blood assay to investigate the polyclonal T cell release of IL17-A after activation with anti-CD3 + anti-CD28 + eventually IL2. In this assay cell separation procedures are avoided by diluting heparinized blood with culture medium, and T cells are activated by specific anti-CD3/antiCD28 antibodies during culture in this plasma/medium mixture as described below (see the chapter "Cell culture"). The in vitro assay and the patient characteristics have been described in detail in a previous publication [13]. Secondly, we investigated the IL17-A release by a panel of T cell clones derived from patients with therapy-induced cytopenia; these patients and the preparation and characterization of the clones have also been described previously [14-16].

### Healthy controls

The controls (n = 30; median age 49.5 years with variation range 21-64 years; 16 male and 14 female) did not differ significantly with regard to age and sex from the patients tested for T cell subset distribution during chemotherapy-induced cytopenia. However, the controls were younger than the patients with untreated disease. Before statistical comparisons we therefore investigated whether there was any age-dependent difference among the controls, and if this was detected we compared untreated patients with those controls being above 50 years of age (n = 15).

### Preparation of enriched AML cells

Leukemic peripheral blood mononuclear cells (PBMC) were isolated by density gradient separation (Ficoll-Hypaque; NyCoMed, Oslo, Norway; specific density 1.077) from the peripheral blood of patients with a high percentage of AML blasts among blood leukocytes (> 85%). Cells were cryopreserved in 20% heat-inactivated fetal calf serum (FCS) plus 10% dimethylsulphoxide (DMSO) and stored in liquid nitrogen. The studies of T cell subsets included 16 unselected patients (median age 62.5 years with variation range 24-82 years; 7 males and 9 females). These cells are also referred to as primary human AML cells (> 95% leukemia cells). Leukemic PBMC were also used for analysis of T cell responsiveness after stimulation with anti-CD2 + anti-CD3 as described below for the whole blood assay.

### Flow cytometry

#### Intracellular IL17-A, IFNγ and FoxP3 in T cells

Sodium Heparin blood samples were obtained and erythrocytes lysed by BD Pharm Lyse solution (BD Biosciences, Trondheim, Norway). Cryopreserved leukemic PBMC were also analyzed. Pre-warmed X-Vivo 10^® ^culture medium (BioWhittaker, Cambridge, MA, USA) was added and the cell concentration adjusted to 10^6 ^cells/mL before incubation for 4 hours with or without 2 μL/mL of Leukocytes Activation cocktail with BD GolgiPlug (BD Biosciences; PMA/Ionomycin/BFA) at 37°C in a humidified atmosphere of 5% CO_2_. The cells were thereafter washed in ice-cold 1% BSA/PBS followed by staining with PerCP-conjugated mouse anti-human CD3 (SK7; BD Biosciences), FITC-conjugated anti-CD8 (RPA-T8; BD Biosciences), FITC-conjugated anti-CD4 (RPA-T4; BD Pharmingen) and/or PE-conjugated anti-CD25 (M-A251; BD Biosciences) on ice for 20 minutes. Samples were washed once in PBS before fixation and permeabilization using eBioscience Fixation/Permeabilization Concentrate (cat. 00-5123), Fixation/Permeabilization Diluent (cat. 00-5223) and Permeabilization Buffer (cat. 00-8333) (eBioscience, San Diego, USA) as recommended by the manufacturer. Intracellular staining was performed at room temperature for 30 minutes with the mouse monoclonal Alexa Fluor 647-conjugated anti-human IL17-A (eBio64DEC17; eBioscience), Alexa Fluor 647-conjugated anti-FoxP3 (259D/C7, BD Biosciences), PE-conjugated anti-IL17-A (eBio64CAP17; eBioscience) or PE-conjugated anti-IFNγ (B27; BD Biosciences). Four-color flow cytometry was performed by a FACS Calibur System. For each sample at least 20 000 lymphocytes were counted. Results were analyzed by FlowJo software (Tree Star, Inc., OR, USA).

#### Estimation of absolute lymphocyte counts

Absolute count determination was done according to the manufacturer's specifications. Briefly, samples were incubated with anti-human CD3-PerCP (BD) for 20 minutes before addition of Flow-Count Fluorospheres (Beckman Coulter, London, UK), followed by immediate analysis by a FACS Calibur System. For each sample 5000 beads were counted. Results were analyzed by FlowJo software (Tree Star).

#### Analysis of IL17 receptor (IL17-R) expression by primary human AML cells

Cells were stained with unconjugated mouse anti-human IL17-R (clone 133617; R&D systems, Abingdon, UK) using QIFIKIT (Dako, Glostrup, Denmark) in combination with anti-CD34 conjugated with APC (BD Biosciences). The analysis was performed according to the manufacturer's instructions. Gates for viable leukemia cells were based on forward and side scatter and 20 000 events were collected.

#### Analysis of STAT, p38, CREB, Erk1/2 and Akt phosphorylation in primary AML cells

As described previously [17], AML cells were incubated for 5 minutes with or without IL17-A, GM-CSF or IFN-γ before STAT, p38, CREB, Erk1/2 and Akt phosphorylation were analyzed by using Alexa647-conjugated anti-phospho-Stat5 (pY694), anti-phospho-Erk1/2 (pT202/pY204) and anti-phospho-Akt (pS473); and Alexa488-conjugated anti-phospho-Stat3 (pY705), anti-phospho-p38 (pT180/pY182) and anti-phospho-Creb (pS133) monoclonal antibodies (BD Biosciences).

### Cell culture

#### Whole blood assay for analysis of IL17-A release

The standardization of this in vitro assay has been described in detail in a previous methodological publication [18], and its use for analysis of T cell cytokine responses in patients with chemotherapy-induced cytopenia has also been described previously [13]. Briefly, heparinized blood samples were diluted with growth medium; cells were thereafter incubated in this plasma/medium mixture and T cells activated with anti-CD3 and anti-CD28 (The Central Laboratory of the Netherlands Red Cross Blood Transfusion Services, Amsterdam, The Netherlands). The T cell concentration in the cultures was < 20 × 10^4^/mL. Cultures were incubated for 4 days at 37°C in a humidified atmosphere of 5% CO_2 _before supernatants were harvested and IL17-A levels determined (Quantikine ELISA kits, R&D Systems). Even though several leukocyte subsets are present in these cultures, it should be emphasized that in our experiments we compared differences in cytokine levels for cultures with and without T cell activation signals (anti-CD3 and anti-CD28), and the most likely explanation for increased cytokine levels after T cell directed signaling is increased release by activated T cells.

#### Proliferation studies of primary human AML cells

This in vitro assay has been described previously [19]. Briefly, 5 × 10^4 ^cells/well were incubated in flat-bottomed microtiter plates (Costar 3796; Cambridge, MA, USA) in 150 μl Stem Span SFEM™ medium (Stem Cell Technologies Inc, Vancouver, BC, Canada) supplemented with 100 μg/mL of gentamicin and eventually 50 ng/ml of IL17, IL1RA, IL1β, IL3, Flt3L, SCF, GM-CSF or G-CSF (PeproTech EC Ltd., London, UK). Proliferation was assayed as ^3^H-thymidine incorporation after 7 days.

### Statistical analysis

The SPSS software was used to perform Wilcoxon's test for paired samples, Mann-Whitney test for unpaired samples, Spearman's rho for correlation or linear bivariate regression analysis.

## Results

### Relative levels of CD3^+ ^T cells are increased in AML patients with therapy-induced cytopenia

**Figure 1:**
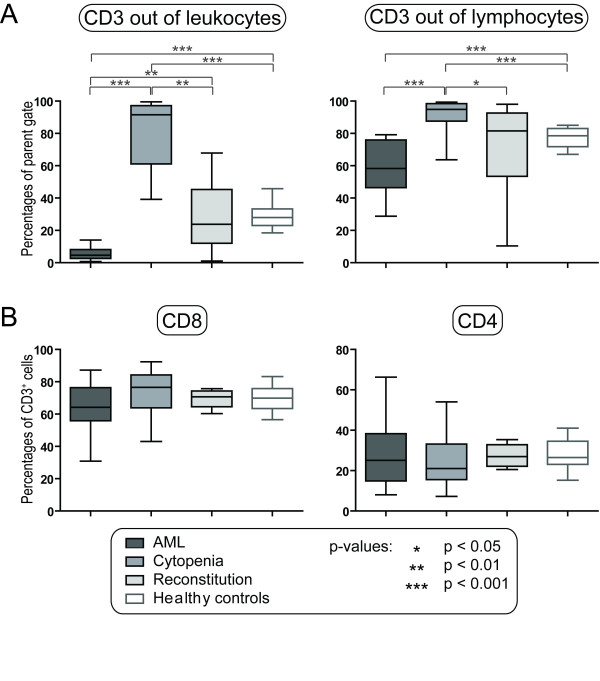
**Relative levels of total T cells and T cell subsets (CD3^+^CD8^+ ^and CD3^+^CD4^+^) in AML patients receiving intensive chemotherapy**. T cell levels were investigated for patients with untreated AML (AML, n = 16), patients with chemotherapy-induced cytopenia (Cytopenia, n = 16; the first cohort of patients) and during hematopoietic reconstitution after chemotherapy (Reconstitution, n = 6); these results were compared with healthy controls (n = 30). **A**. The levels of circulating CD3^+ ^T cells are presented as the percentages of total viable leukocytes (left panel) or total lymphocytes (right panel). **B**. The levels CD8^+ ^(T_C_) and CD4^+ ^(i.e. CD3^+^CD8^- ^cells referred to as CD4^+ ^T_H _cells) T cell levels are presented as the percentages of positive cells among total CD3^+ ^lymphocytes. All results are presented as median (horizontal line), 25^th ^to 75^th ^percentiles (boxes) and variation range (whiskers). The percentages of total T cells or T cell subsets did not show any age-dependent variation among the healthy controls. Statistically significant differences are indicated at the top of the figures (Mann-Whitney test).

We investigated the levels of circulating CD3^+ ^T cells for patients with untreated AML (n = 16) and leukemia patients with severe chemotherapy-induced cytopenia (n = 16) and during reconstitution after chemotherapy (n = 6). Patients were compared with healthy controls (n = 30) and the overall results are presented in Figure 1A. As expected the percentage of CD3^+ ^T cells among total leukocytes was decreased and depended on the degree of leukemization for patients with untreated disease. During chemotherapy-induced cytopenia significantly increased levels of circulating CD3^+ ^T cells were observed (generally > 60%; median level 91.5%), and very few normal (monocytes, granulocytes) and leukemic myeloid cells remained. During hematopoietic reconstitution (i.e. circulating neutrophils > 0.5 × 10^9 ^/L) the percentage of CD3^+ ^T cells showed an expected normalization although a wide variation was observed among the patients.

We then compared the relative CD3^+ ^T cell levels among total lymphocytes in the patient groups (Figure 1A). For some untreated patients the lymphocytes and AML cells had overlapping side and forward scatter (for a detailed discussion see [20]), and these patients (5/16) had to be excluded from this analysis. Patients with chemotherapy-induced cytopenia showed significantly increased percentages of CD3^+ ^cells among total lymphocytes compared to healthy controls and patients with untreated disease. The CD3^+ ^percentage thereafter normalized during hematopoietic reconstitution, although the variation range was wider than for the controls.

### The relative levels of CD8^+ ^and CD4^+ ^T cells are normal following intensive chemotherapy

The levels of circulating CD8^+ ^cytotoxic (T_C_) and CD8^- ^(referred to as CD3^+ ^CD4^+^) helper (T_H_) T cells among total CD3^+ ^T cells were determined in all three patient groups (Figure 1B). The T_C _and T_H _levels showed only minor variations in the AML patients and did not differ significantly from the healthy controls. However, both for T_C _and T_H _cells the variation ranges were generally wider for patients with untreated disease and severe chemotherapy-induced cytopenia than for the patients in the reconstitution phase and for healthy controls.

### Circulating T_C_1 and T_H_1 cells are decreased during severe chemotherapy-induced cytopenia

**Figure 2:**
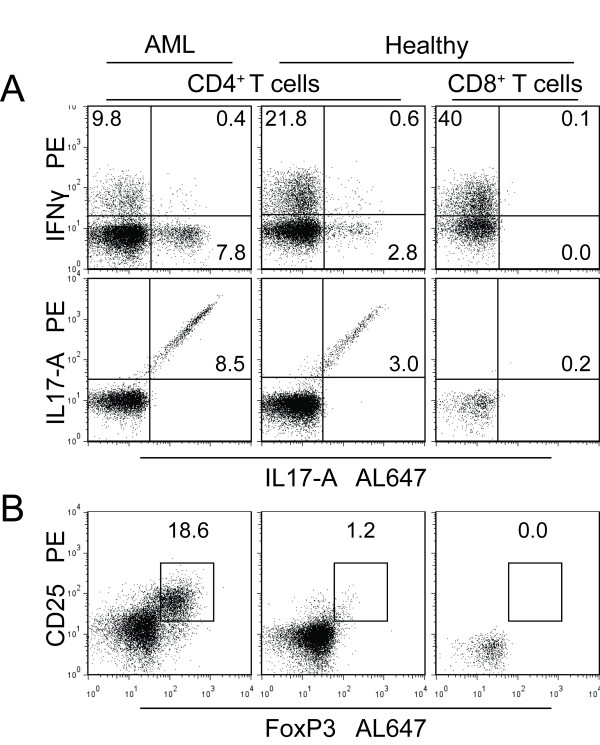
**Strategies for flow-cytometric analysis of intracellular IL17-A, IFNγ and FoxP3**. Peripheral blood lymphocytes were incubated for 4 hours with PMA/Ionomycin/BFA (IL17 and IFNγ) or in medium alone (FoxP3) followed by intracellular staining and analysis by flow cytometry. **A**. IFNγ and IL17-A accumulation in CD8^- ^(referred to as CD4^+ ^cells) after 4 hours of stimulation with PMA/Ionomycin/BFA. The detection of IL17-A in CD8^- ^(CD4^+^) T cells was verified by using two different antibodies. **B**. The levels of regulatory T cells were analyzed by detection of CD4^+ ^CD25^+ ^FoxP3^+ ^cells. For both panels CD3^+ ^CD8^+ ^T cells were used as negative intra-sample controls for both T_H_17 and T_REG _cells.

**Figure 3:**
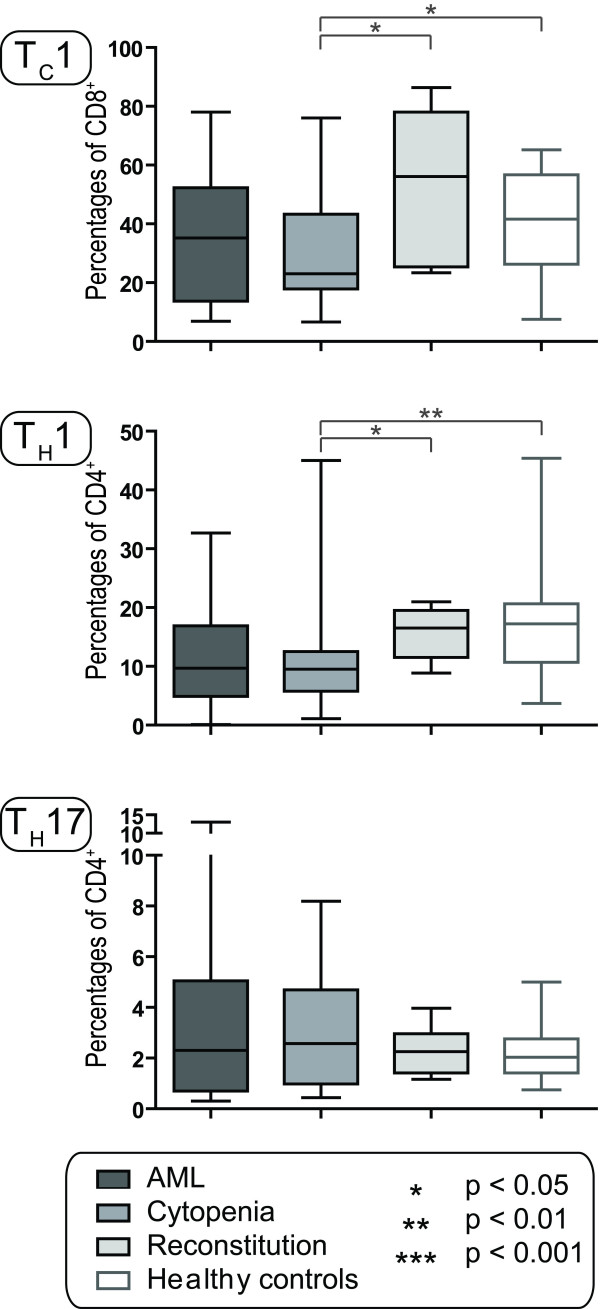
**The T_C_1, T_H_1 and T_H_17 subsets in AML patients and healthy controls**. The levels of circulating T_C_1, T_H_1 and T_H_17 cells were investigated for patients with untreated AML (AML, n = 16), patients with chemotherapy-induced cytopenia (Cytopenia, n = 16; the first cohort of patients) and during hematopoietic reconstitution after chemotherapy (Reconstitution, n = 6); these results were compared with healthy controls (n = 30). The levels for each of the subsets are presented as the percentage of each cell type among the parent population; i.e. T_C_1 cells among CD8^+ ^cells, T_H_1 and T_H_17 cells among CD8^- ^T cells and T_REG _cells among CD4^+ ^T cells. All results are presented as median (horizontal line), 25^th ^to 75^th ^percentiles (boxes) and variation range (whiskers). The percentages of total T cells or T cell subsets did not show any age-dependent variation among the healthy controls. Statistically significant differences are presented at the top of the figure (Mann-Whitney test).

Patients were investigated for relative levels of circulating T_C_1 and T_H_1 cells among CD3^+ ^T cells by intracellular IFNγ staining. Gating strategies are shown in Figure 2A (upper part) and the overall results for patients and healthy controls are presented in Figure 3. Relative levels of T_C_1 cells, defined by the expression of CD3^+ ^CD8^+ ^and IFNγ^+^, were normal in untreated AML. However, the levels were decreased during chemotherapy-induced cytopenia (median level 23%, range 7-76%; p = 0.046) compared to the controls (median level 41.6%, range 8-65%) before normalization during reconstitution (median level 56%, range 23-86%). Relative levels of T_H_1 cells, defined by the phenotype CD3^+ ^CD8^- ^IFNγ^+^, were also significantly decreased during therapy-induced cytopenia (median level 9.5%, range 1-45%) compared to the controls (median level 17%, range 4-45%) before normalization occurred during reconstitution (median level 16.5%, range 9-21%). Finally, the absolute levels of T_C_1 and T_H_1 cells were also decreased and significantly correlated when investigated in the second cohort of patients (p < 0.005).

### Relative levels of circulating T_H_17 cells are maintained in AML patients with chemotherapy-induced cytopenia

The patients were examined for relative levels of circulating T_H_17 cells by intracellular staining against IL17-A followed by flow cytometric analysis. Gating strategies are shown in Figure 2A (lower part) and the overall results for patients and healthy controls are presented in Figure 3. The relative levels of circulating T_H_17 cells, defined by the phenotype CD3^+ ^CD8^- ^IL17-A^+^, did not differ between healthy controls and AML patients with untreated disease, treatment-induced cytopenia and regeneration after chemotherapy. However, patients with untreated disease and chemotherapy-induced cytopenia showed wider variation ranges than the other groups.

We examined levels of IL17-A in culture supernatants when T cells among leukemic PBMC (n = 17, T cell concentration in cultures < 2 × 10^4^/mL) were activated with anti-CD3 plus anti-CD28 as described for the whole blood assay. Detectable IL17-A levels up to 90 pg/mL were observed after T cell activation; this is similar to normal PBMC (T cell concentration 2-3 × 10^5^/mL) that also showed detectable release up to 87 pg/mL (data not shown). Thus, because this IL17-A release occurred in response to T cell specific stimulation (anti-CD3 + anti-CD28) the most likely explanation for the increased levels is that the ability of circulating T cells to release IL17-A is maintained in the presence of primary AML cells even though these cells show constitutive release of several immunoregulatory cytokines [21]. IL17 levels are actually higher for untreated patients than for the controls when considering the different T cell concentrations in the cultures.

We investigated the polyclonal T cell release of IL17-A for another group of AML patients with chemotherapy-induced cytopenia [13]; peripheral blood leukocytes were then cultured in a whole blood assay and IL17-A levels determined in the culture supernatants (see Additional file 1: Figure S1). Samples cultured in medium alone or with isotypic control antibodies usually showed undetectable (23 out of 25 samples) or low IL17-A levels (< 37 pg/mL). Detectable levels were present only for a minority of samples (6/25) after stimulation with anti-CD3 + anti-CD28 and anti-CD3 + anti-CD28 + IL2, but these levels were significantly higher than for the corresponding unstimulated control cultures (p = 0.027). An unselected subset of 17 consecutive samples was in addition cultured with the antileukemic drug Pep005 [13,22] in combination with anti-CD3 + anti-CD28 + IL2. Pep005 significantly increased the IL17-A levels compared with the corresponding drug-free controls, and detectable levels were then observed for 11 of the 17 samples (see Additional file 1: Figure S1). Finally we compared IL17-A levels with the levels of IFNγ, GM-CSF, IL2, IL3, IL4, IL5, IL6, IL10, IL13 and TNFα after T cell activation with anti-CD3 + anti-CD28 and anti-CD3 + anti-CD28 + IL2. GM-CSF was the only cytokine that showed statistically significant correlations with IL17-A both after stimulation with anti-CD3 + anti-CD28 (p < 0.001, rho = 0.5) and anti-CD3 + anti-CD28 + IL2 (p = 0.001, rho = 0.5). Thus, the remaining T_H_17 cells in cytopenic patients are functional and because these increased levels occurred after T cell-specific stimulation with anti-CD3 + anti-CD28 the most likely explanation is that the remaining T cells are able to release detectable IL17-A in response to adequate stimulation as a part of a broad cytokine response.

We compared phytohemagglutinin and anti-CD3 stimulated IL17-A release by a panel of T cell clones derived from another group of cytopenic patients [14-16]. Detectable IL17 release was observed for 5 out of 51 CD4^+ ^CD8^- ^T cell clones and 2 out of 36 CD4^- ^CD8^+ ^clones. These results further confirmed that IL17-A-releasing T cells (i.e. T_H_17 cells) remain in the circulation during severe chemotherapy-induced cytopenia. However, the IL17-A levels were relatively low (15- 26 pg/ml) for those clones with detectable release. All the IL17-A releasing T cell clones were CD2^+ ^CD3^+ ^CD19^- ^TCRαβ^+ ^TCRγδ^- ^CD57^- ^and additionally released IFNγ and GM-CSF (data not shown).

Thus, only a minority of remaining clonogenic T cells is able to release detectable IL17-A and the frequencies are comparable to those detected by the flow cytometry assay.

### Relative levels of circulating CD4^+ ^CD25^+ ^FoxP3^+ ^T cell are increased in AML patients before and following intensive chemotherapy

**Figure 4:**
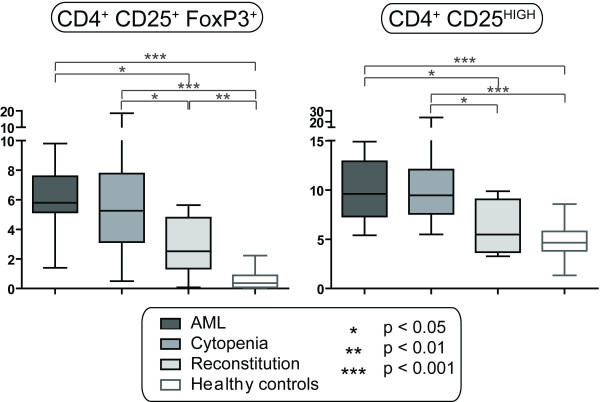
**Circulating FoxP3^+ ^T_REG _cells and CD4^+ ^CD25^HIGH ^T cells in AML patients and healthy controls**. The levels of CD4^+ ^FoxP3^+ ^T_REG _cells and CD3^+ ^CD4^+ ^CD25^HIGH ^T cells were analyzed for patients with untreated AML (AML, n = 16), patients with chemotherapy-induced cytopenia (Cytopenia, n = 16; the first cohort of patients) and during hematopoietic reconstitution after chemotherapy (Reconstitution, n = 6); these results were compared with healthy controls (n = 30). The levels are presented as the percentage of each cell type among CD4^+ ^T cells. No age-dependent differences were observed in the control group. All results are presented as median (horizontal line), 25^th ^to 75^th ^percentiles (boxes) and variation range (whiskers). Statistically significant differences are presented at the top of the figures (Mann-Whitney test).

The relative levels of circulating T_REG _cells were determined for all the patients by intracellular staining against FoxP3 and analysis by flow cytometry. Gating strategies are shown in Figure 2B and the overall results are presented in Figure 4. Increased levels of natural T_REG _cells, defined by the phenotype CD3^+ ^CD4^+ ^CD25^+ ^FoxP3^+^, were detected for AML patients with untreated disease, chemotherapy-induced cytopenia and regeneration after treatment when compared with healthy controls. The T_REG _levels were highest for patients with untreated disease and chemotherapy-induced cytopenia; thereafter the levels decreased significantly but were still higher than for the controls. As an alternative strategy for analysis of T_REG _cells we determined the percentage of CD3^+^CD4^+^CD25^HIGH ^cells in all the samples (Figure 4). The analysis confirmed that the highest levels of T_REG _cells were detected in patients with untreated leukemia and during chemotherapy-induced cytopenia (Figure 4). The FoxP3^+ ^and CD25^HIGH ^T_REG _levels were significantly correlated both in patients with untreated leukemia and during chemotherapy-induced cytopenia (p < 0.001).

### Relative levels of circulating T_H_17 and T_H_1 cells show significant correlations in AML patients with untreated disease and treatment-induced cytopenia

**Figure 5:**
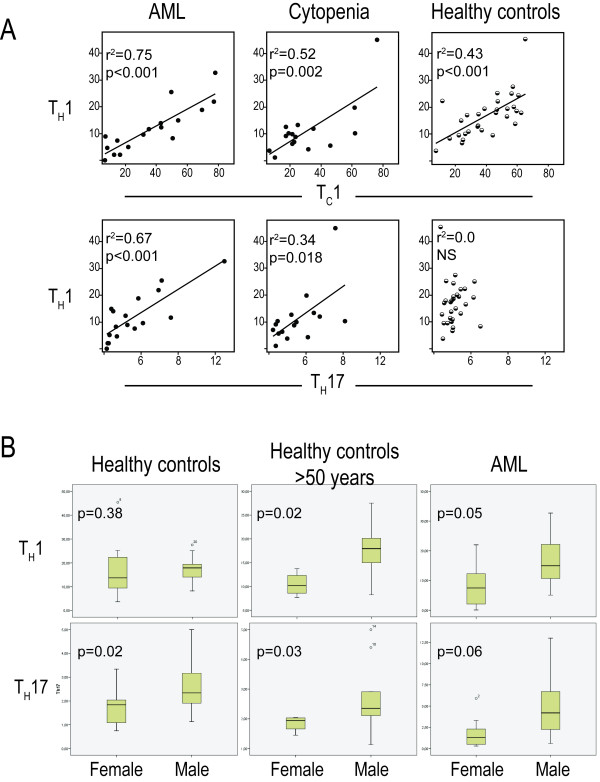
**Peripheral blood levels of T_H_1 and T_H_17 cells; analysis of covariation between various T cell subsets and differences between males and females**. **A**. Bivariate regression analysis was used to analyze the correlations between T_H_1/T_C_1 and T_H_1/T_H_17 cell subsets in AML patients with untreated disease (AML, n = 16) and patients with chemotherapy-induced cytopenia (Cytopenia, n = 16; the first cohort of patients); these results were compared with healthy controls (n = 30). The levels are presented as the percentage of each cell type among the parent population, i.e. T_C_1 cells among CD8^+ ^T cells, T_H_1 and T_H_17 cells among CD8^- ^T cells. The p- and r^2^-values for each analysis are indicated in each figure. **B**. Analysis of T_H_1 and T_H_17 levels in male and female healthy controls (all controls and those controls older than 50 years) and patients with untreated AML patients. The levels are presented as the percentage of each cell type among CD8^- ^T cells. All results are presented as the median (line within the box), 25^th ^to 75^th ^percentiles (box) and variation ranges (whiskers), are presented as percentages of parent. P-values are given in each figure.

We compared the levels of circulating T_C_1, T_H_1, T_H_17 and T_reg _cells for patients and healthy controls (Figure 5A). Significant correlations between the levels of circulating T_H_17 and T_H_1 cells were detected for AML patients with untreated disease and during chemotherapy-induced cytopenia; this correlation was stronger for untreated (r^2 ^= 0.67) than for cytopenic patients (r^2 ^= 0.34) and no correlation was found during reconstitution and for in healthy controls. Even though we also found significant correlations between T_H_1 and Tc1 levels for all groups except patients in reconstitution (Figure 5A), the T_H_17 and T_C_1 levels showed no correlation (data not shown). Finally, T_REG _cells showed no significant correlations with any other T cell subset (data not shown). These observations were also confirmed when investigating absolute T cell counts in the second cohort of patients; T_H_17 and T_H_1 levels then showed a highly significant correlation (p < 0.001), whereas T_REG _cells showed no significant correlations with any other T cell subset.

### T_H_17 and T_H_1 levels are significantly higher in healthy males than females but this gender-dependent difference is not detected after intensive AML chemotherapy

Significantly higher levels of circulating T_H_17 cells were observed for healthy males (Figure 5B; n = 16, median levels 2.3% range 1.1-5.0%) compared with females (n = 14; median 1.9%, range 0.8-3.3%; p = 0.02). This difference was detected both for younger and elderly controls. A similar difference was also observed when comparing male (n = 7; median 4.2%, range 0.6-7.2) and female (n = 9; median 1.0, range 0.3-3.3) patients with untreated AML, although it reached only borderline significance (p = 0.06). In contrast, T_H_1 levels differed between males and females when comparing the elderly controls above 50 years of age but not when comparing the whole control group; a similar difference of borderline significance (Figure 5B; p = 0.05) was observed only for patients with untreated disease. No gender differences in T_H_17 and T_H_1 levels were seen for AML patients during cytopenia or reconstitution (data not shown). Finally, the levels of circulating T cell subsets showed no significant correlations with cytogenetics, induction or consolidation chemotherapy or serum level of C reactive protein at the time of sampling.

### Acute leukemia patients with severe chemotherapy-induced pancytopenia: studies of absolute levels of various T cell subsets in a second cohort of patients

We investigated relative and absolute T cell levels in a second cohort of 8 consecutive AML patients receiving intensive chemotherapy. For all patients the relative T_REG _levels were increased during cytopenia (median level 10%, range 7-32%) compared with pretherapy levels (median level 3%, range 2-16%) and the levels detected during early reconstitution (median 7%, range 5-13%). The absolute T_REG _cell counts showed a wide variation between the patients during cytopenia (median counts 14.1 cells/μl, range 5.6-59.8), and this wide variation persisted during the early hematopoietic reconstitution with increasing neutrophil counts (median counts 24.6 cells/μl, range 6.8-60.8). For two of these patients the absolute T_REG _counts increased markedly during cytopenia compared with the pretherapy and early regeneration levels; levels up to 59.8 cells/μL were then observed and this increase was not accompanied with an increase of total CD4^+ ^T cells. In contrast to T_REG _levels, the absolute T_H_17 cell counts showed only minor variations in individual patients during cytopenia; the counts were within the range of 3-10.9 cells/μL, and for 3 of the 8 patients stable counts below 5 cells/μl were detected. Pretherapy levels were generally within the same range (1.1-17.5 cells/μl), whereas the absolute T_H_17 levels showed a wider variation during early reconstitution (median counts 13.4 cells/μl, range 1.0-35.0). Thus, these results support the data from the first patient cohort: absolute and relative levels of circulating T_REG _cells are increased during cytopenia whereas the T_H_17 levels only shows minor variations after chemotherapy.

**Figure 6:**
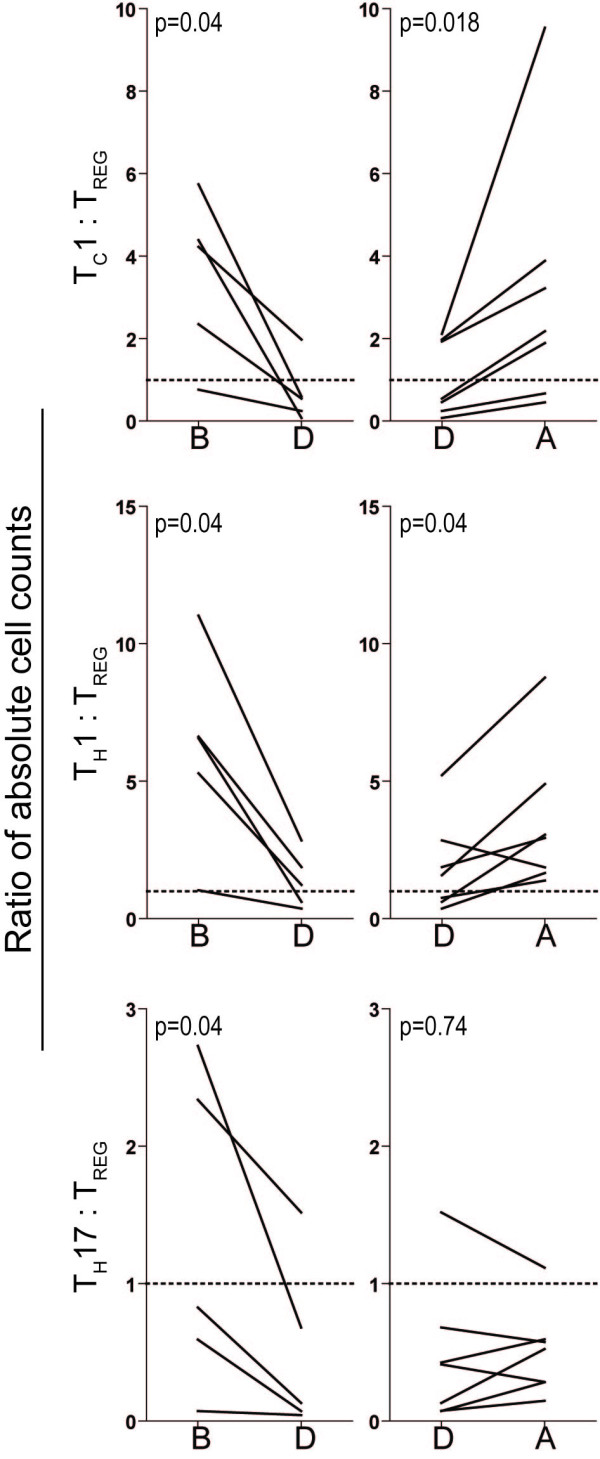
**Peripheral blood T cell levels in AML patients receiving intensive chemotherapy; T_C_1:T_REG_, T_H_1:T_REG _and T_H_17:T_REG _ratios determined before, during and following severe treatment-induced cytopenia**. The T_C_1:T_REG_, T_H_1:T_REG _and T_H_17:T_REG _ratios were determined before (B), during (D) and after (A) severe chemotherapy-induced cytopenia for patients included in the second cohort. All results are presented as absolute cell concentration in peripheral blood (cells per μL blood) for T_C_1, T_H_1 or T_H_17 cells relative to the corresponding levels of T_REG _cells. Each line indicates the results for one patient, and P-values are given in each figure (Wilcoxon for paired samples).

We also compared the T_C_1:T_REG_, T_H_1:T_REG _and T_H_17:T_REG _ratios for patients tested before, during and following severe chemotherapy-induced cytopenia (Figure 6). All ratios decreased during cytopenia, i.e. the levels of the three other subsets decreased compared with the T_REG _levels. Finally, when comparing the overall results T_REG _and T_H_17 absolute cell counts showed no statistically significant correlation; this is similar to the observations in the first cohort.

### IL17-A has only minor effects on the functional characteristics of primary human AML cells

T_H_17 cells and IL17 have important immunoregulatory functions but also an additional regulatory role in normal hematopoiesis [23]. Furthermore, previous studies have also demonstrated that IL17-RB can be expressed by t(8;21)-positive AML cells [24]. For these reasons we investigated whether IL17 had a direct role in regulation of leukemic hematopoiesis; this has been described for several other T cell derived cytokines [25-27]. First we investigated IL17-R expression by primary AML cells derived from 13 consecutive patients. For 10 of these patients we detected a small IL17-R^+ ^CD34^- ^leukemic subpopulation (see Additional file 2: Figure S2; median level of IL17-R^+ ^leukemic cells being 9%, variation range 1.5 - 34.7%); and none of these patients expressed the genetic t(8;21) abnormality. Furthermore, when comparing the overall results for the 13 patients we did not observe any significant alteration in the phosphorylation status of intracellular Stat3, Stat5, p38, Erk1/2, Creb and Akt after IL17-R ligation, although responses were observed for some individual patients (see Additional file 2: Figure S2). An expected intracellular phosphoresponse was observed after exposure to GM-CSF and IFNγ (data not shown).

We also investigated whether IL17-A had any effects on AML cell proliferation. The overall results are shown in Additional file 3: Table S1. Leukemic cells derived from 59 consecutive patients were cultured in vitro with and without IL17-A 50 ng/mL. Detectable spontaneous proliferation corresponding to at least 1000 counts per minute (cpm) was observed only for 13/59 patients, and IL17-A caused a significant enhancement of the in vitro proliferation (median 6386 cpm; interquartile range 11.826 cpm: p = 0.002). The leukemic cells were also cultured with and without IL17-A in the presence of 50 ng/mL of IL1RA, IL1β, IL3, Flt3L, SCF, G-CSF or GM-CSF. IL17-A caused a minor, though statistically significant, increase of cytokine-dependent proliferation in the presence of IL1β, IL3, SCF, G-CSF and GM-CSF. However, the increase exceeded 2000 cpm and corresponded to > 20% of the IL17-A-free control only for a small minority of patients. IL17-A had no significant effect on clonogenic AML cell proliferation and did not affect the constitutive AML cell release of IL1β, IL6 and TNFα (data not shown). To summarize, IL17A-R is expressed only by a minority of primary AML cells, receptor ligation usually does not induce detectable intracellular phosphoresponses and exogenous IL17-A has only minor effects on functional AML cell characteristics.

## Discussion

Previous studies of T_REG _cells in human AML have included patients with untreated disease and patients in complete hematological remission. AML patients with chemotherapy-induced cytopenia have not been examined, even though clinical studies suggest that immunological events early after chemotherapy are important for the antileukemic effect of chemotherapy. In the present study we therefore compared T cell subset distribution in AML patients with severe chemotherapy-induced cytopenia with (i) untreated patients or patients during regeneration after intensive chemotherapy; and (ii) healthy controls. The cytopenic patients had increased relative levels of circulating T_REG _cells, whereas levels of T_C_1 and T_H_1 cells were decreased while T_H_17 cells were not altered.

Early lymphoid reconstitution after chemotherapy is associated with decreased risk of leukemia relapse [4-7,28], and these observations suggest that immunological events early after chemotherapy are clinically important. The immunological status during treatment-induced cytopenia are determined by pre-existing disease-induced abnormalities and chemotherapy-induced defects [21]. Previous studies have demonstrated that even patients with severe therapy-induced lymphopenia have an operative T cell system [21], and in this context we investigated the balance between various proinflammatory and suppressive T cell subsets in AML patients.

As expected the relative levels of CD3^+ ^T lymphocytes to total leukocytes were dependent on the degree of leukemization in patients with untreated AML. More important, the frequencies of circulating CD3^+ ^T cells among total lymphocytes were decreased in patients with untreated disease compared with healthy controls, these levels were increased during chemotherapy-induced pancytopenia before normalization during reconstitution. Even though our cytopenic patients had lymphopenia, our results suggest that CD3^+ ^T cells are less sensitive to intensive chemotherapy than myeloid cells and other lymphocyte subsets (e.g. B cells, NK cells).

We did not find any significant differences in relative CD8^+ ^and CD4^+ ^T cell levels between healthy controls and patients examined before or following intensive chemotherapy. In contrast, the relative levels of circulating IFNγ secreting CD4^+ ^(T_H_1) and CD8^+ ^(T_C_1) T cells were decreased in cytopenic patients before normalization during reconstitution (Figure 3). This decrease is also reflected in the decreased T_C_1:T_REG _and T_H_1:T_REG _ratios during cytopenia (Figure 6). Taken together these observations strongly suggest that treatment-induced lymphopenia is not a random process and the susceptibility to intensive chemotherapy differ between T cell subsets.

T_H_17 cells constitute a separate proinflammatory T helper (T_H_) cell subset [29-33]. Studies in animals as well as humans suggest that T_H_17 cells are important for anticancer immune reactivity [34,35]. A previous study described increased frequencies of circulating T_H_17 cells in patients with untreated AML and with a normalization when patients achieved complete remission after chemotherapy [11]. In contrast, we observed normal T_H_17 levels for patients with untreated AML. In addition we observed that functional T_H_17 cells could be detected during severe therapy-induced cytopenia, and these frequencies did not differ from healthy controls although the variation range was broader. Studies of absolute and relative T_H_17 levels in a second patient cohort confirmed that only minor variations were seen during therapy-induced cytopenia. The decreased T_H_17:T_REG _ratio during cytopenia is thus caused by relatively stable T_H_17 absolute levels with only minor decreases and maintained or increasing absolute levels of T_REG _cells (Figure 6). Finally, we observed increased IL17-A release by circulating cells in response to stimulation by T cell specific antibodies (anti-CD3 combined with anti-CD28); this is probably caused by T cell release because IL17-A release from other cells in response to these T cell specific stimulatory signals seems less likely. Our overall results thereby strongly suggest that neither the leukemia nor the chemotherapy has any selective suppressive effect on T_H_17 cells compared with other T cell subsets. Release of IL17-A from other cells than T cells activated in response to these stimulatory signals seems less likely.

Immunosuppressive CD4^+ ^T_REG _cells inhibit effector T cells and NK cells [36,37]. Depletion of T_REG _cells enhances murine anti-cancer immunity [38,39] as well as vaccine-induced anticancer reactivity in humans [40]. Increased levels of circulating T_REG _cells seem to occur in untreated AML [9,10], although these previous studies did not analyze FoxP3^+ ^cells. We observed increased levels of circulating FoxP3^+ ^T_REG _cells in patients with untreated AML. Even though previous studies suggest that at least certain cytotoxic drugs can reduce the levels of circulating T_REG _cells [41], our present results showed that the frequencies of T_REG _cells were increased before and following intensive AML chemotherapy. Studies in a second cohort of patients demonstrated that the absolute levels of Treg cells even increased during cytopenia for certain patients. This was true even for patients regenerating after therapy, an observation that is also supported by another study [9]. The relative levels of CD4^+ ^CD25^HIGH ^T_REG _cell were also increased in untreated AML and during cytopenia. The variations of various T cell subsets relative to T_REG _cells then suggest that the various subsets are differentially affected, and the increased T_REG _levels may then be caused either by T_REG _proliferation or development from T cell progenitors or other T cell subsets.

We observed a correlation between the levels of T_H_1 and T_C_1 cells both for AML patients and healthy controls, and this correlation was maintained during severe therapy-induced cytopenia. In contrast, the correlation between T_H_1 and T_H_17 levels was detected only for AML patients with untreated disease and therapy-induced cytopenia. These observations further support the hypothesis that various T cell subsets are differentially affected in AML patients.

The levels of circulating T_H_17 cells were significantly higher in males than females for healthy controls, and T_H_1 cells were also higher in males for elderly controls. In a recent gene expression study of activated T cells the responsiveness was generally higher in women than in men, but IL17-A was the only effector gene that showed highest expression in men [42]. Estrogen response elements in the promoter regions of several immune genes could possibly explain the gender differences [42]. This gender differences reached only borderline statistical significance for patients with untreated AML and could not be detected in our cytopenic patients. Thus, the influence of gender differences on circulating T cell subsets becomes less important after chemotherapy.

Exogenous IL17-A had no or only weak effects on functional AML cell characteristics; even though some differences were statistically significant their biological significance can be questioned. Even though the relative levels of T_H_17 cells are maintained after chemotherapy, we conclude that T_H_17 cells may affect AML cells indirectly through their immunoregulatory effects; whereas direct effects of IL17-A on the leukemic cells probably do not have any major impact.

## Conclusions

To conclude, AML patients have an altered distribution of circulating T cell subsets; this is due to both disease-associated and chemotherapy-induced alterations whereas gender-dependent differences in T_H_1 and T_H_17 levels become less important after intensive chemotherapy. Our results suggest that T_REG _cells are relatively resistant to intensive AML therapy as increased levels persist even during the period of severe cytopenia. Previous studies suggest a prognostic impact of T_REG _cells in AML patients receiving intensive chemotherapy, but our present results suggest that the altered T_REG _levels are only a part of a more complex immunological phenotype in these patients. Taken together our studies strongly suggest that the possible prognostic impact of variations in circulating T cell subsets should be addressed in future clinical studies.

## List of Abbreviations

AML: acute myeloid leukemia; G-CSF: granulocyte colony-stimulating factor; GM-CSF: granulocyte macrophage colony-stimulating factor; IFNγ: interferon gamma; IL: interleukin; IL17-R: IL17 receptor; n: numbers; NK: natural killer; PBMC: peripheral blood mononuclear cells; SCF: stem cell factor; T_C_: cytotoxic T cells; T_H_1: helper T cells type 1; T_H_17: helper T cells type 17; T_REG_: regulatory T cells; WHO: world health organization.

## Authors' contributions

The work presented here was carried out in collaboration between all authors. EE and ØB defined the research theme. EE designed methods and experiments, carried out the laboratory experiments, analyzed the data, interpreted the results and wrote the paper. EE, JS and BTG co-designed the phosphoflow experiments, and co-worked on the associated data collection and their interpretation. KL had responsibility of the patients' medical journals, tables, interpretation, and presentation. ØB contributed in all aspects of the study. All authors have contributed to, seen and approved the manuscript.

## Supplementary Material

Additional file 1 **Release of IL17-A by T cells derived from AML patients with chemotherapy-induced cytopenia**. Peripheral blood leukocytes were cultured in the whole blood assay and IL17-A levels determined in the culture supernatants. The leukocytes were cultured in medium alone (CTR), or medium with aCD3 + aCD28 or aCD3 + aCD28 + IL2. A subset of samples were also added the PKC agonist Pep005 in combination with aCD3 + aCD28 + IL2 (denoted Pep005). Results are presented as the cytokine concentration for each sample. Grey circles represent levels below the minimum detectable concentration (< 15 pg/mL), and black circles represent detectable levels. Click here for file

Additional file 2 **IL17-A receptor expression and phosphorylation of intracellular mediators following IL17-A stimulation**. Primary leukemia cells derived from 13 untreated AML patients (AML 1-13) were assessed for IL17-A receptor (IL17-R) expression (grey area) by flow cytometry. The lower part of the figures shows the percentages of IL17-R^+ ^cells out of total viable leukemia cells (IL17-R %) and mean fluorescence intensity (MFI) of the IL17-R positive cells (MFI+) versus the IL17-R negative cells (MFI-). The primary human AML cells were also stimulated (Stim), or not stimulated (Unstim), with IL17-A for 5 minutes before cells were assessed for phosphorylation status of Stat3, Stat5, p38, Erk, Creb and Akt. The results are presented as histogram overlay of unstimulated and stimulated samples with color designating fold change (-0.5 - 0.5). Click here for file

Additional file 3 **The effect of IL17-A on spontaneous and cytokine-dependent AML cell proliferation, a summary of the results for 59 consecutive patients**. Primary human AML cells were cultured in serum-free medium alone, with IL17-A, or with IL17-A in combination with the indicated exogenous cytokines. AML cell proliferation was assayed as 3H-thymidine incorporation after 7 days of culture *in vitro*. Click here for file

## References

[B1] Smith M Barnett M Bassan R Gatta G Tondini C Kern W Adult acute myeloid leukaemia Crit Rev Oncol Hematol 2004 50 197 222 10.1016/j.critrevonc.2003.11.002 15182826

[B2] Williams KM Hakim FT Gress RE T cell immune reconstitution following lymphodepletion Semin Immunol 2007 19 318 30 10.1016/j.smim.2007.10.004 18023361 PMC2180244

[B3] Auletta JJ Lazarus HM Immune restoration following hematopoietic stem cell transplantation: an evolving target Bone Marrow Transplant 2005 35 835 57 10.1038/sj.bmt.1704966 15778723

[B4] Behl D Porrata LF Markovic SN Letendre L Pruthi RK Hook CC Tefferi A Elliot MA Kaufmann SH Mesa RA Absolute lymphocyte count recovery after induction chemotherapy predicts superior survival in acute myelogenous leukemia Leukemia 2006 20 29 34 10.1038/sj.leu.2404032 16281063

[B5] Porrata LF Litzow MR Tefferi A Letendre L Kumar S Geyer SM Markovic SN Early lymphocyte recovery is a predictive factor for prolonged survival after autologous hematopoietic stem cell transplantation for acute myelogenous leukemia Leukemia 2002 16 1311 8 10.1038/sj.leu.2402503 12094255

[B6] Parkman R Cohen G Carter SL Weinberg KI Masinsin B Guinan E Kurtzberg J Wagner JE Kernan NA Successful immune reconstitution decreases leukemic relapse and improves survival in recipients of unrelated cord blood transplantation Biol Blood Marrow Transplant 2006 12 919 27 10.1016/j.bbmt.2006.05.008 16920557

[B7] Kim DH Sohn SK Won DI Lee NY Suh JS Lee KB Rapid helper T-cell recovery above 200 × 10 6/l at 3 months correlates to successful transplant outcomes after allogeneic stem cell transplantation Bone Marrow Transplant 2006 37 1119 28 10.1038/sj.bmt.1705381 16699530

[B8] Obeid M Tesniere A Panaretakis T Tufi R Joza N van Endert P Ghiringhelli F Apetoh L Chaput N Flament C Ecto-calreticulin in immunogenic chemotherapy Immunol Rev 2007 220 22 34 10.1111/j.1600-065X.2007.00567.x 17979837

[B9] Szczepanski MJ Szajnik M Czystowska M Mandapathil M Strauss L Welsh A Foon KA Whiteside TL Boyiadzis M Increased frequency and suppression by regulatory T cells in patients with acute myelogenous leukemia Clin Cancer Res 2009 15 3325 32 10.1158/1078-0432.CCR-08-3010 19417016 PMC3700356

[B10] Wang X Zheng J Liu J Yao J He Y Li X Yu J Yang J Liu Z Huang S Increased population of CD4(+)CD25(high), regulatory T cells with their higher apoptotic and proliferating status in peripheral blood of acute myeloid leukemia patients Eur J Haematol 2005 75 468 76 10.1111/j.1600-0609.2005.00537.x 16313258

[B11] Wu C Wang S Wang F Chen Q Peng S Zhang Y Qian J Jin J Xu H Increased frequencies of T helper type 17 cells in the peripheral blood of patients with acute myeloid leukaemia Clin Exp Immunol 2009 158 199 204 10.1111/j.1365-2249.2009.04011.x 19737137 PMC2768809

[B12] Vardiman JW Harris NL Brunning RD The World Health Organization (WHO) classification of the myeloid neoplasms Blood 2002 100 2292 302 10.1182/blood-2002-04-1199 12239137

[B13] Ersvaer E Hampson P Hatfield K Ulvestad E Wendelbo O Lord JM Gjertsen BT Bruserud O T cells remaining after intensive chemotherapy for acute myelogenous leukemia show a broad cytokine release profile including high levels of interferon-gamma that can be further increased by a novel protein kinase C agonist PEP005 Cancer Immunol Immunother 2007 56 913 25 10.1007/s00262-006-0236-5 17115221 PMC11030909

[B14] Bruserud O Ulvestad E Cytokine responsiveness of mitogen-activated T cells derived from acute leukemia patients with chemotherapy-induced leukopenia J Interferon Cytokine Res 2000 20 947 54 10.1089/10799900050198381 11096451

[B15] Bruserud O Ulvestad E Berentsen S Bergheim J Nesthus I T-lymphocyte functions in acute leukaemia patients with severe chemotherapy-induced cytopenia: characterization of clonogenic T-cell proliferation Scand J Immunol 1998 47 54 62 10.1046/j.1365-3083.1998.00254.x 9467659

[B16] Bruserud O Ulvestad E Acute myelogenous leukemia blasts as accessory cells during in vitro T lymphocyte activation Cell Immunol 2000 206 36 50 10.1006/cimm.2000.1725 11161436

[B17] Irish JM Hovland R Krutzik PO Perez OD Bruserud O Gjertsen BT Nolan GP Single cell profiling of potentiated phospho-protein networks in cancer cells Cell 2004 118 217 28 10.1016/j.cell.2004.06.028 15260991

[B18] Wendelbo O Bruserud O Functional evaluation of proliferative T cell responses in patients with severe T lymphopenia: characterization of optimal culture conditions and standardized activation signals for a simple whole blood assay J Hematother Stem Cell Res 2003 12 525 35 10.1089/152581603322448231 14594509

[B19] Bruserud O Ryningen A Wergeland L Glenjen NI Gjertsen BT Osteoblasts increase proliferation and release of pro-angiogenic interleukin 8 by native human acute myelogenous leukemia blasts Haematologica 2004 89 391 402 15075072

[B20] Ersvaer E Hampson P Wendelbo O Lord JM Gjertsen BT Bruserud O Circulating T cells in patients with untreated acute myelogenous leukemia are heterogeneous and can be activated through the CD3/TCR complex Hematology 2007 12 199 207 10.1080/10245330701255163 17558695

[B21] Ersvaer E Olsnes AM Bruserud O The Immunological Dilemma: Cellular Innate and Adaptive Immune Response Versus Human Acute Myeloid Leukemia Open Hematology Reviews 2007 1 1 14

[B22] Hampson P Chahal H Khanim F Hayden R Mulder A Assi LK Bunce CM Lord JM PEP005, a selective small-molecule activator of protein kinase C, has potent antileukemic activity mediated via the delta isoform of PKC Blood 2005 106 1362 8 10.1182/blood-2004-10-4117 15845901

[B23] Schwarzenberger P Kolls JK Interleukin 17: an example for gene therapy as a tool to study cytokine mediated regulation of hematopoiesis J Cell Biochem Suppl 2002 38 88 95 10.1002/jcb.10054 12046855

[B24] Tonks A Pearn L Musson M Gilkes A Mills KI Burnett AK Darley RL Transcriptional dysregulation mediated by RUNX1-RUNX1T1 in normal human progenitor cells and in acute myeloid leukaemia Leukemia 2007 21 2495 505 10.1038/sj.leu.2404961 17898786

[B25] Ersvaer E Skavland J Ulvestad E Gjertsen BT Bruserud O Effects of interferon gamma on native human acute myelogenous leukaemia cells Cancer Immunol Immunother 2006 10.1007/s00262-006-0159-1 PMC11030278 16612597

[B26] Stone RM Mayer RJ Treatment of the newly diagnosed adult with de novo acute myeloid leukemia Hematol Oncol Clin North Am 1993 7 47 64 8449864

[B27] Beran M Andersson B Kantarjian H Keating M Rios A McCredie KB Freireich EJ Gutterman J Hematologic response of four patients with smoldering acute myelogenous leukemia to partially pure gamma interferon Leukemia 1987 1 52 7 2444829

[B28] Kim H Sohn HJ Kim S Lee JS Kim WK Suh C Early lymphocyte recovery predicts longer survival after autologous peripheral blood stem cell transplantation in multiple myeloma Bone Marrow Transplant 2006 37 1037 42 10.1038/sj.bmt.1705373 16708062

[B29] Chen Z JJ O'Shea Th17 cells: a new fate for differentiating helper T cells Immunol Res 2008 41 87 102 10.1007/s12026-007-8014-9 18172584

[B30] Steinman L A brief history of T(H)17, the first major revision in the T(H)1/T(H)2 hypothesis of T cell-mediated tissue damage Nat Med 2007 13 139 45 10.1038/nm1551 17290272

[B31] Bettelli E Carrier Y Gao W Korn T Strom TB Oukka M Weiner HL Kuchroo VK Reciprocal developmental pathways for the generation of pathogenic effector TH17 and regulatory T cells Nature 2006 441 235 8 10.1038/nature04753 16648838

[B32] Mangan PR Harrington LE O'Quinn DB Helms WS Bullard DC Elson CO Hatton RD Wahl SM Schoeb TR Weaver CT Transforming growth factor-beta induces development of the T(H)17 lineage Nature 2006 441 231 4 10.1038/nature04754 16648837

[B33] Awasthi A Murugaiyan G Kuchroo VK Interplay between effector th17 and regulatory T cells J Clin Immunol 2008 28 660 70 10.1007/s10875-008-9239-7 18810613

[B34] Kryczek I Wei S Zou L Altuwaijri S Szeliga W Kolls J Chang A Zou W Cutting edge: Th17 and regulatory T cell dynamics and the regulation by IL-2 in the tumor microenvironment J Immunol 2007 178 6730 3 17513719 10.4049/jimmunol.178.11.6730

[B35] Le Gouvello S Bastuji-Garin S Aloulou N Mansour H Chaumette MT Berrehar F Seikour A Charachon A Karoui M Leroy K High prevalence of Foxp3 and IL17 in MMR-proficient colorectal carcinomas Gut 2008 57 772 9 10.1136/gut.2007.123794 17965063

[B36] Piccirillo CA Shevach EM Cutting edge: control of CD8+ T cell activation by CD4+CD25+ immunoregulatory cells J Immunol 2001 167 1137 40 11466326 10.4049/jimmunol.167.3.1137

[B37] Sakaguchi S Takahashi T Yamazaki S Kuniyasu Y Itoh M Sakaguchi N Shimizu J Immunologic self tolerance maintained by T-cell-mediated control of self-reactive T cells: implications for autoimmunity and tumor immunity Microbes Infect 2001 3 911 8 10.1016/S1286-4579(01)01452-6 11564439

[B38] Shimizu J Yamazaki S Sakaguchi S Induction of tumor immunity by removing CD25+CD4+ T cells: a common basis between tumor immunity and autoimmunity J Immunol 1999 163 5211 8 10553041

[B39] Litzinger MT Fernando R Curiel TJ Grosenbach DW Schlom J Palena C IL-2 immunotoxin denileukin diftitox reduces regulatory T cells and enhances vaccine-mediated T-cell immunity Blood 2007 110 3192 201 10.1182/blood-2007-06-094615 17616639 PMC2200901

[B40] Dannull J Nair S Su Z Boczkowski D DeBeck C Yang B Gilboa E Vieweg J Enhancing the immunostimulatory function of dendritic cells by transfection with mRNA encoding OX40 ligand Blood 2005 105 3206 13 10.1182/blood-2004-10-3944 15618466

[B41] Bruserud O Ersvaer E Olsnes A Gjertsen BT Anticancer Immunotherapy in Combination with Proapoptotic Therapy Curr Cancer Drug Targets 2008 8 666 675 10.2174/156800908786733496 19075589

[B42] Hewagama A Patel D Yarlagadda S Strickland FM Richardson BC Stronger inflammatory/cytotoxic T-cell response in women identified by microarray analysis Genes Immun 2009 10 509 16 10.1038/gene.2009.12 19279650 PMC2735332

