# Supplementary material for: Intensive chemotherapy for acute myeloid leukemia differentially affects circulating TC1, TH1, TH17 and TREG cells
Source: BMC Immunol. 2010 Jul 9;11:38. doi: 10.1186/1471-2172-11-38 (PMC2912832; doi:10.1186/1471-2172-11-38)
Supplement: Additional file 1 — Release of IL17-A by T cells derived from AML patients with chemotherapy-induced cytopenia. Peripheral blood leukocytes were cultured in the whole blood assay and IL17-A levels determined in the culture supernatants. The leukocytes were cultured in medium alone (CTR), or medium with aCD3 + aCD28 or aCD3 + aCD28 + IL2. A subset of samples were also added the PKC agonist Pep005 in combination with aCD3 + aCD28 + IL2 (denoted Pep005). Results are presented as the cytokine concentration for each sample. Grey circles represent levels below the minimum detectable concentration (< 15 pg/mL), and black circles represent detectable levels. [file 1471-2172-11-38-S1.DOC]

**Additional file 1: Figure S1**


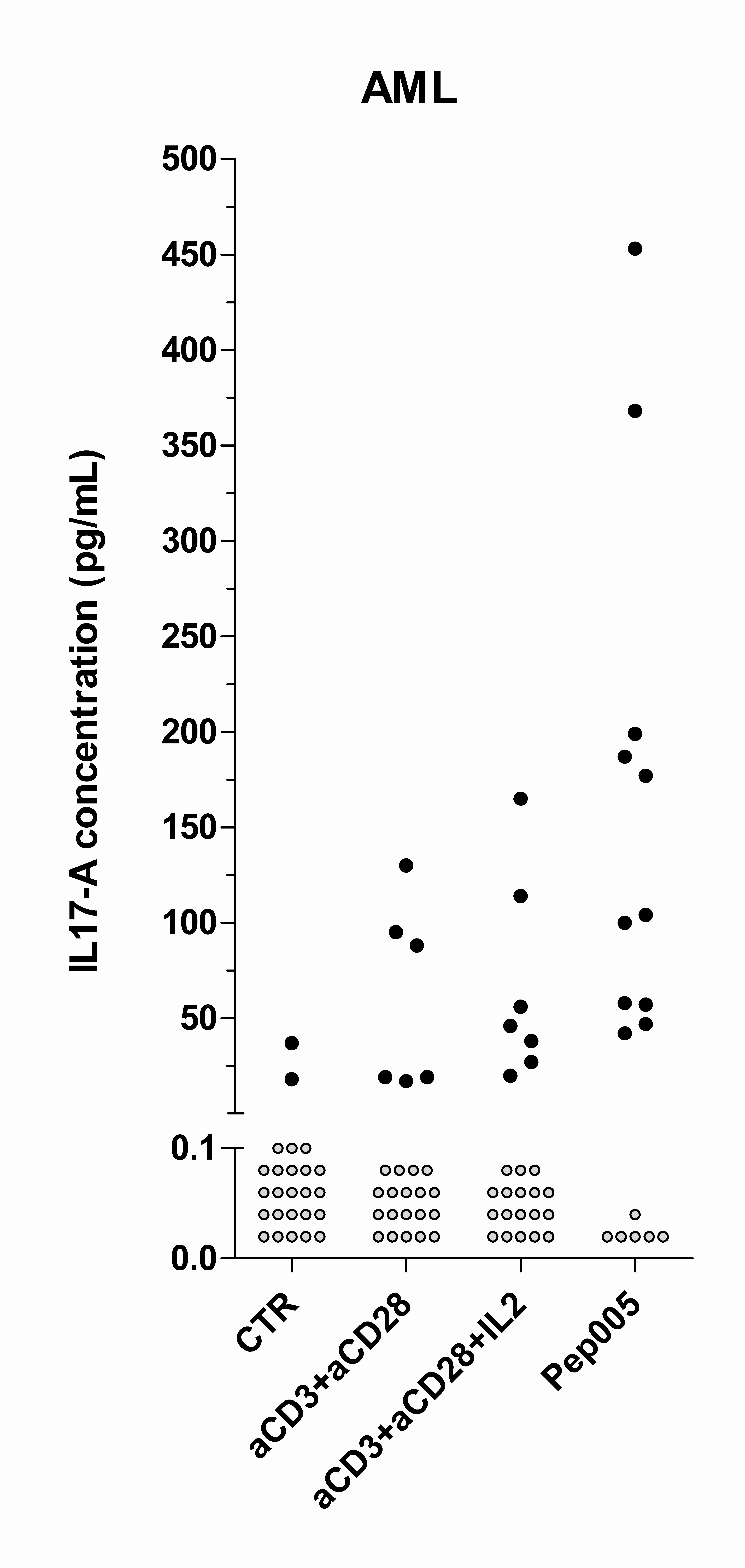
Release of IL17-A by T cells derived from AML patients with chemotherapy-induced cytopenia. Peripheral blood leukocytes were cultured in the whole blood assay and IL17-A levels determined in the culture supernatants. The leukocytes were cultured in medium alone (CTR), or medium with aCD3 + aCD28 or aCD3 + aCD28 + IL2. A subset of samples were also added the PKC agonist Pep005 in combination with aCD3 + aCD28 + IL2 (denoted Pep005). Results are presented as the cytokine concentration for each sample. Grey circles represent levels below the minimum detectable concentration (<15 pg/mL), and black circles represent detectable levels.
