# Supplementary material for: Intensive chemotherapy for acute myeloid leukemia differentially affects circulating TC1, TH1, TH17 and TREG cells
Source: BMC Immunol. 2010 Jul 9;11:38. doi: 10.1186/1471-2172-11-38 (PMC2912832; doi:10.1186/1471-2172-11-38)

Additional file 2: Figure S2

IL17-A receptor expression and phosphorylation of intracellular mediators following IL17-A stimulation. Primary leukemia cells derived from 13 untreated AML patients (AML 1-13) were assessed for IL17-A receptor (IL17-R) expression (grey area) by flow cytometry. The lower part of the figures shows the percentages of IL17-R^+^ cells out of total viable leukemia cells (IL17-R %) and mean fluorescence intensity (MFI) of the IL17-R positive cells (MFI+) versus the IL17-R negative cells (MFI-). The primary human AML cells were also stimulated (Stim), or not stimulated (Unstim), with IL17-A for 5 minutes before cells were assessed for phosphorylation status of Stat3, Stat5, p38, Erk, Creb and Akt. The results are presented as histogram overlay of unstimulated and stimulated samples with color designating fold change (-0.5 – 0.5).


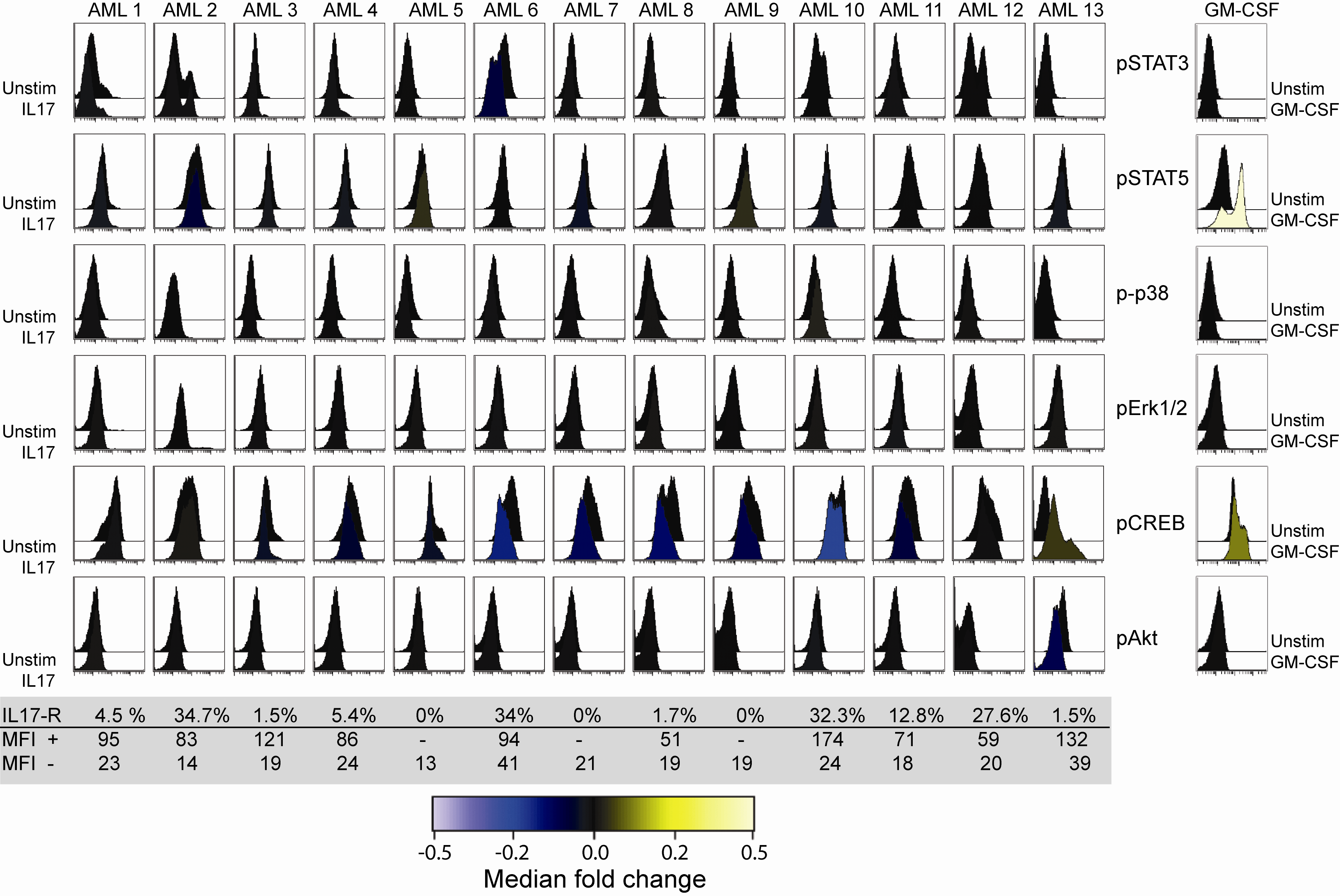

Supplement: Additional file 2 — IL17-A receptor expression and phosphorylation of intracellular mediators following IL17-A stimulation. Primary leukemia cells derived from 13 untreated AML patients (AML 1-13) were assessed for IL17-A receptor (IL17-R) expression (grey area) by flow cytometry. The lower part of the figures shows the percentages of IL17-R+ cells out of total viable leukemia cells (IL17-R %) and mean fluorescence intensity (MFI) of the IL17-R positive cells (MFI+) versus the IL17-R negative cells (MFI-). The primary human AML cells were also stimulated (Stim), or not stimulated (Unstim), with IL17-A for 5 minutes before cells were assessed for phosphorylation status of Stat3, Stat5, p38, Erk, Creb and Akt. The results are presented as histogram overlay of unstimulated and stimulated samples with color designating fold change (-0.5 - 0.5). [file 1471-2172-11-38-S2.DOC]
