# Supplementary material for: Intensive chemotherapy for acute myeloid leukemia differentially affects circulating TC1, TH1, TH17 and TREG cells
Source: BMC Immunol. 2010 Jul 9;11:38. doi: 10.1186/1471-2172-11-38 (PMC2912832; doi:10.1186/1471-2172-11-38)
Supplement: Additional file 3 — The effect of IL17-A on spontaneous and cytokine-dependent AML cell proliferation, a summary of the results for 59 consecutive patients. Primary human AML cells were cultured in serum-free medium alone, with IL17-A, or with IL17-A in combination with the indicated exogenous cytokines. AML cell proliferation was assayed as 3H-thymidine incorporation after 7 days of culture in vitro. [file 1471-2172-11-38-S3.DOC]

Additional file 3: Table S1

The effect of IL17-A on spontaneous and cytokine-dependent AML cell proliferation, a summary of the results for 59 consecutive patients.a

|  |  |  |  |  |  | |  |  |  |
| --- | --- | --- | --- | --- | --- | --- | --- | --- | --- |
|  |  |  |  | Statistical comparison of proliferative respons | | | |  | Number of samples  with >20% enhancement and absolute increase >2000 cpm ^d^ |
| Exogenous  cytokine |  | Number of samples with  detectable proliferation^b^ |  | median cpm (interquartile range) | | | |  |  |
|  |  |  |  | Cultures without IL17-A | | Cultures with IL17-A | p-value^c^ |  |  |
| None |  | 18 |  | 1 938 (6 309) | | 6 386 (11 826) | 0.002 |  | 6/18 |
| IL1RA |  | 24 |  | 3 734 (9 238) | | 4 718 (6 789) | ns |  | 3/24 |
|  |  |  |  |  | |  |  |  |  |
| IL1β |  | 40 |  | 2 772 (7 744) | | 5 075 (11 830) | <0.001 |  | 14/40 |
| IL3 |  | 43 |  | 9 021 (19 793) | | 11 253 (20 329) | 0.006 |  | 7/43 |
| SCF |  | 48 |  | 9 444 (28 040) | | 10 721 (27 707) | 0.027 |  | 11/48 |
| Flt3L |  | 47 |  | 8 384 (15 900) | | 8 075 (16 441) | ns |  | 4/47 |
| GM-CSF |  | 39 |  | 7 180 (13 985) | | 8 042 (18 558) | 0.001 |  | 6/39 |
| G-CSF |  | 45 |  | 6 396 (16 705) | | 6 981 (18 496) | <0.001 |  | 8/45 |
|  |  |  |  |  | |  |  |  |  |

1. Primary human AML cells were cultured in serum-free medium and proliferation assayed as ^3^H-thymidine incorporation after 7 days. The results are presented as count per minute (cpm).
2. A total of 59 consecutive patient samples were examined, but only those samples with detectable proliferation (>1000 cpm) either in the IL17-containing or the corresponding IL17-A-free control were included in the statistical analysis.
3. The two-tailed Wilcoxon's signed rank test was used for the statistical analysis. Not significant: ns.
4. These results are presented as the number of samples with an IL17-A-induced alteration corresponding to at least 2000 cpm and exceeding 20% of the control response relative to the total number of samples with detectable proliferation.
